# Heterogeneous Vascular Bed Responses to Pulmonary Titanium Dioxide Nanoparticle Exposure

**DOI:** 10.3389/fcvm.2017.00033

**Published:** 2017-05-24

**Authors:** Alaeddin B. Abukabda, Phoebe A. Stapleton, Carroll R. McBride, Jinghai Yi, Timothy R. Nurkiewicz

**Affiliations:** ^1^Department of Physiology and Pharmacology, West Virginia University School of Medicine, Morgantown, WV, USA; ^2^Department of Pharmacology and Toxicology, Rutgers University, Piscataway, NJ, USA

**Keywords:** engineered nanomaterials, titanium dioxide, cardiovascular system, microcirculation, endothelium

## Abstract

A growing body of research links engineered nanomaterial (ENM) exposure to adverse cardiovascular endpoints. The purpose of this study was to evaluate the impact of ENM exposure on vascular reactivity in discrete segments so that we may determine the most sensitive levels of the vasculature where these negative cardiovascular effects are manifest. We hypothesized that acute nano-TiO_2_ exposure differentially affects reactivity with a more robust impairment in the microcirculation. Sprague-Dawley rats (8–10 weeks) were exposed to nano-TiO_2_ *via* intratracheal instillation (20, 100, or 200 µg suspended per 250 µL of vehicle) 24 h prior to vascular assessments. A serial assessment across distinct compartments of the vascular tree was then conducted. Wire myography was used to evaluate macrovascular active tension generation specifically in the thoracic aorta, the femoral artery, and third-order mesenteric arterioles. Pressure myography was used to determine vascular reactivity in fourth- and fifth-order mesenteric arterioles. Vessels were treated with phenylephrine, acetylcholine (ACh), and sodium nitroprusside. Nano-TiO_2_ exposure decreased endothelium-dependent relaxation in the thoracic aorta and femoral arteries assessed *via* ACh by 53.96 ± 11.6 and 25.08 ± 6.36%, respectively. Relaxation of third-order mesenteric arterioles was impaired by 100 and 20 µg nano-TiO_2_ exposures with mean reductions of 50.12 ± 8.7 and 68.28 ± 8.7%. Cholinergic reactivity of fourth- and fifth-order mesenteric arterioles was negatively affected by nano-TiO_2_ with diminished dilations of 82.86 ± 12.6% after exposure to 200 µg nano-TiO_2_, 42.6 ± 12.6% after 100 µg nano-TiO_2_, and 49.4 ± 12.6% after 20 µg nano-TiO_2_. Endothelium-independent relaxation was impaired in the thoracic aorta by 34.05 ± 25% induced by exposure to 200 µg nano-TiO_2_ and a reduction in response of 49.31 ± 25% caused by 100 µg nano-TiO_2_. Femoral artery response was reduced by 18 ± 5%, while third-order mesenteric arterioles were negatively affected by 20 µg nano-TiO_2_ with a mean decrease in response of 38.37 ± 10%. This is the first study to directly compare the differential effect of ENM exposure on discrete anatomical segments of the vascular tree. Pulmonary ENM exposure produced macrovascular and microvascular dysfunction resulting in impaired responses to endothelium-dependent, endothelium-independent, and adrenergic agonists with a more robust dysfunction at the microvascular level. These results provide additional evidence of an endothelium-dependent and endothelium-independent impairment in vascular reactivity.

## Introduction

Engineered nanomaterials (ENMs) are most commonly defined as a homogeneous mixture of anthropogenic materials possessing at least one dimension less than or equal to 100 nm (1) and have historically been used in inhalation toxicology research as surrogates for environmental air pollution studies to model ultrafine particulate matter exposures (2, 3). The continued development of novel ENM with diverse physicochemical properties represents a growing economic sector. Presently, the nanotechnology industry is one of the fastest growing markets with an estimated projected worth of $75.8 billion by 2020 (4). This increased prevalence of ENM not only continues concerns over their safety and the potential adverse health outcomes that may occur as a result of prolonged, unmitigated exposure but also warrants more thorough toxicological assessments of their potential deleterious systemic effects.

Titanium dioxide (nano-TiO_2_) is among the most widely used nanomaterials, and its optical properties allow it to be used in consumer products such as topical sunscreens and cosmetics, as well as industrial components including paints (5). It is used commonly as a photocatalyst, catalyst carrier, heat stabilizer, and food additive (6). Nano-TiO_2_ may also be used as a drug carrier in nanomedicine (7).

Pulmonary nano-TiO_2_ exposure has been associated with systemic inflammation *in vivo* (8–10), with dose dependent overt inflammation characterized by a marked increase in proinflammatory mediators and lung toxicity (10, 11).

The circulatory system can be grossly divided into two major components based on structure and location: the macrocirculation and the microcirculation. The macrocirculation refers to the conduit arteries and veins, which connect the heart with the systemic organs and also function as pressure and blood reservoirs, respectively. The microcirculation is a broad term encompassing all the vascular tissue within a specific organ, including arterioles, the capillary network, and venules (12). Its fundamental function is the maintenance of an ideal internal environment for the controlled exchange of nutrients and metabolic waste with the adjacent tissues. For this study, conduit arteries and arterioles were chosen because they are major hemodynamic regulators and key players in the maintenance of blood pressure, flow distribution, and tissue perfusion (13, 14).

The endothelium is a regulator of vascular homeostasis and is extremely sensitive to changes in blood composition and hemodynamics (15). Consisting of a monolayer of cells that functions as a physical barrier for the exchange of materials between blood and tissues (16), it is also responsible for the secretion of a variety of molecules, including prostacyclins (17), clotting factors (18), and ectonucleotidases (19) that are important for the regulation of blood coagulation and platelet function. Endothelial cells also coordinate the recruitment of immune cells to sites of injury or infection *via* both the production and release of cytokines (20) and the expression of specific cell adhesion molecules on their apical surface that mediate leukocyte attachment and extravasation (21–24). While common to all vascular segments, endothelial cells show significant morphological and functional heterogeneity throughout the vasculature, between different organs, and between neighboring endothelial cells of the same organ and blood vessel type (25). These phenotypic differences may account for the differential and vascular bed-specific responses of endothelial cells to specific toxicants.

We have previously established that nano-TiO_2_ exposure is associated with systemic vascular dysfunction (26–28). However, the relative sensitivity to ENM of each vascular segment is not known. Therefore, the aim of this study is to assess the effects of three occupationally relevant nano-TiO_2_ concentrations on both the macrocirculation and the microcirculation from a functional perspective *via* wire and pressure myography. One hundred micrograms of nano-TiO_2_ were selected based on previous work conducted by our group (2). In addition, 200 and 20 µg were chosen for this study to identify the maximum and minimum observed dose responses. The most sensitive vascular level to nano-TiO_2_ exposure was subsequently determined. On the basis of our previous results, we hypothesize that nano-TiO_2_ exposure impairs endothelium-dependent responsiveness and that this effect is more pronounced in the microcirculation.

## Materials and Methods

### Nanomaterial Characterization

Nano-TiO_2_ P25 powder, obtained from Evonik (Aeroxide TiO_2_, Parsippany, NJ, USA), has previously been shown to be a mixture composed primarily of anatase (80%) and rutile (20%) TiO_2_, with a primary particle size of 21 nm and a surface area of 48.08 m^2^ g^−1^ (2, 29, 30).

### Experimental Animals and Exposure

Male (8–10 weeks) Sprague–Dawley rats were purchased from Hilltop Laboratories (Scottdale, PA, USA) and housed at WVU with 12:12 h light–dark cycle and regulated temperature. Rats were allowed *ad libitum* access to food and water. All procedures were approved by the Institutional Animal Care and Use Committee of West Virginia University and abide by the standards set forth in the “Guide for Care and Use of Laboratory Animals” of the National Research Council of the National Academies.

Bolus doses of nano-TiO_2_ (20, 100, and 200 µg) were suspended in 250 µL of vehicle (normosol and 5% fetal bovine serum) for intratracheal instillation (IT) 24 h prior to experimentation. Nano-TiO_2_ suspensions were sonicated over ice for 1 min to ensure increased dispersion. Sprague-Dawley rats were anesthetized using 5% isoflurane and placed on a mounting stand for IT with 200 µL of vehicle (negative control) or the nano-TiO_2_ suspensions.

### Mean Arterial Pressure (MAP) Acquisition

Rats were anesthetized with isoflurane gas (5% induction, 3–3.5% maintenance) and placed on a heating pad to maintain a 37°C rectal temperature. The trachea was intubated to ensure an open airway, and the right carotid artery was cannulated to acquire MAP. PowerLab830 (AD Instruments) was used to record MAP.

### Wire Myography Arterial Ring Preparation

The thoracic aorta, common femoral artery, and third-order mesenteric arterioles were dissected and immediately placed in ice cold Ringer’s solution. Two- to three-mm segments were cut and mounted in each wire myograph chamber (AD Instruments—DMT 620 M) containing 5 mL of physiological salt solution (PSS; 119 mM NaCl, 4.7 mM KCl, 1.18 mM KH_2_PO_4_, 1.17 mM MgSO_4_, 2.5 mM CaCl_2_, 25 mM NaHCO_3_, 0.027 mM EDTA, and 5.5 mM glucose) at pH 7.4 and bubbled with 95% O_2_/5% CO_2_ at 37°C. After 30 min of equilibration, maximum contractile response was determined by using high-potassium PSS (K + PSS; 123.7 mM KCl, 1.18 mM KH_2_PO_4_, 1.17 mM MgSO_4_, 2.5 mM CaCl_2_, 25 mM NaHCO_3_, 0.027 mM EDTA, and 5.5 mM glucose). Vessels were then washed twice with PSS and allowed to relax until initial tension was reached. Contractile responses were determined *via* cumulative additions of 50 µL of phenylephrine (PE; 1 × 10^−9^ to 1 × 10^−4^ M). Relaxation responses were evaluated *via* cumulative addition of 50 µL acetylcholine (ACh) or sodium nitroprusside (SNP) (1 × 10^−9^ to 1 × 10^−4^ M).

### Statistics

Point-to-point differences in the dose–response curves were evaluated using two-way repeated measures analysis of variance (ANOVA) with a Tukey’s *post hoc* analysis when significance was found. The slopes of the dose–response curves were determined through non-linear regression. The animal characteristics, vessel characteristics, and dose–response curve slopes were analyzed using a one-way ANOVA with a Tukey’s *post hoc* analysis when significance was found. All statistical analysis was completed with GraphPad Prism 5 (San Diego, CA, USA) and SigmaPlot 11.0 (San Jose, CA, USA). Significance was set at *P* < 0.05, *n* is the number of arterioles, and *N* is the number of animals.

### Wire Myography Calculations

Maximum tension was defined as the tension developed by the vessels with K + PSS. Tension with agonists (ACh, SNP, or PE) was recorded, and the percentage of maximum tension generation was calculated using the following formula:

$$\begin{array}{l} \text{Percentage maximum tension (\%)}=\left(\frac{\text{Tension with agonists (mN)}}{\text{Maximum tension (mN)}}\right)\times 100\text{.} \end{array}$$

The response of the different arterial branching orders was quantitatively assessed and compared by using the slope of the individual dose–response curves as previously reported (31).

### Pressure Myography Arteriolar Preparation

The mesentery was excised and placed in a dissecting dish with PSS maintained at 4°C. Fourth- and fifth-order mesenteric arterioles were isolated, transferred to a vessel chamber, cannulated between two glass pipettes (outer diameter 60 µm), and secured by means of silk sutures in the vessel chamber (Living Systems Instrumentation, Burlington, VT, USA). The chamber was superfused with fresh oxygenated (5% CO_2_/21% O_2_) PSS and warmed to 37°C. Arterioles were pressurized to 60 mmHg using a servo control system and extended to their *in situ* length. Internal and external arteriolar diameters were measured using video calipers (Colorado Video, Boulder, CO, USA).

### Arteriolar Reactivity

Arterioles were equilibrated for 1 h prior to experimentation. Arterioles with <20% spontaneous tone were not analyzed. Contractile responses were determined *via* cumulative additions of 50 µL of PE (1 × 10^−9^ to 1 × 10^−4^ M). Relaxation responses were evaluated *via* cumulative addition of 50 µL ACh or sodium nitroprusside (SNP) (1 × 10^−9^ to 1 × 10^−4^ M). The steady-state diameter of the vessel was recorded for at least 2 min after each dose. After each dose curve was completed, the vessel chamber was washed to remove excess chemicals by carefully removing the superfusate and replacing it with fresh warmed oxygenated PSS. After all experimental treatments were completed, the PSS was replaced with Ca^2+^-free PSS until maximum passive diameter was established.

### Pressure Myography Calculations

All measurements were conducted during steady-state conditions, with steady state being defined as the interval during which blood vessels maintained a stable tone for 1 min or more. Vessels were washed twice with PSS between individual dose–response treatments and allowed approximately 10 min for equilibration or until original tone was developed. Data are expressed as means ± SE. Spontaneous tone was calculated by the following equation:

$$\text{Spontaneous tone (}\%\text{)}=\left\{\frac{\left(D_{\text{m}}-D_{\text{i}}\right)}{D_{\text{i}}}\right\}\times 100,$$

where *D*_m_ is the maximal diameter and *D*_i_ is the initial steady-state diameter recorded prior to the experiment. Active responses to pressure were normalized to the maximal diameter using the following formula:

$$\text{Normalized diameter}=D_{\text{ss}}/D_{\text{m}},$$

where *D*_ss_ is the steady-state diameter recorded during each pressure change. The experimental responses to ACh and SNP are expressed using the following equation:

$$\text{Diameter (percent maximal diameter)}=\left\{\frac{\left(D_{\text{ss}}-D_{\text{con}}\right)}{\left(D_{\text{m}}-D_{\text{con}}\right)}\right\}\times 100,$$

where *D*_Con_ is the control diameter recorded prior to the dose curve and *D*_SS_ is the steady-state diameter at each dose of the curve. The experimental response to PE is expressed using the following equation:

$$\text{Diameter (percent maximal diameter)}=\left\{\frac{\left(D_{\text{con}}-D_{\text{ss}}\right)}{\left(D_{\text{con}}\right)}\right\}\times 100.$$

Wall thickness was calculated from the measurement of both inner (ID) and outer (OD) steady-state arteriolar diameters at the end of the Ca^2+^-free wash using the following equation:

WT=(OD−ID)/2.

Wall-to-lumen ratio was calculated using the following equation:

WLR=WT/ID.

## Results

### Animal and Vessel Characteristics

No significant changes were observed in age, MAP, heart rate, and body weight between control and exposure groups (Table 1). Active inner diameter of fourth and fifth order mesenteric arterioles exposed to 200 µg of nano-TiO_2_ (134.3 ± 11 µm) and 100 µg of nano-TiO_2_ (128.3 ± 19 µm) were significantly different from control values (115.4 ± 10 µm), while passive diameter remained unchanged. This increase in diameter was associated with a decreased initial vascular tone (Table 2).

**Table 1:** **Animal characteristics**.

Treatment	Number of rats (*N*)	Age (weeks)	Body weight (g)	Mean arterial pressure (mmHg)	Heart rate (bpm)
Sham control	16	8.5 ± 0.2	288 ± 18	90 ± 4	336 ± 18
Nano-TiO_2_ 200 µg	17	9.3 ± 0.4	295 ± 18	94 ± 4	330 ± 8
Nano-TiO_2_ 100 µg	16	9.5 ± 0.1	319 ± 10	82 ± 3	323 ± 10
Nano-TiO_2_ 20 µg	12	9.4 ± 1.5	322 ± 19	87 ± 3	311 ± 16

*Physical characteristics of control and exposed rats are listed. Values are shown as mean ± SEM. Statistics were analyzed with two-way analysis of variance (*P* ≤ 0.05)*.

*N, number of animals*.

**Table 2:** **Arteriolar characteristics**.

	*n*	Inner diameter (μm)	Outer diameter (μm)	Tone (%)	Passive diameter inner (μm)	Passive diameter outer (μm)	Wall thickness (μm)	Wall-to-lumen ratio	Wall tension (N m^**−**1^)
Sham control	11	115.4 ± 10	139.4 ± 17	31.2 ± 7	183.6 ± 11	152 ± 8	16 ± 3	0.104 ± 0.03	0.61 ± 0.2
Nano-TiO_2_ 200 µg	10	134.3 ± 11*	146.2 ± 15	22.4 ± 4*	187 ± 20	153.7 ± 19	17 ± 1	0.108 ± 0.02	0.62 ± 0.1
Nano-TiO_2_ 100 µg	9	128 ± 19*	144.6 ± 24	23.5 ± 1*	182.5 ± 11	148.7 ± 10	17 ± 3	0.11 ± 0.02	0.6 ± 0.3
Nano-TiO_2_ 20 µg	10	110.7 ± 12	145.9 ± 12	20.3 ± 3	180.4 ± 16	152.6 ± 14	14 ± 2	0.09 ± 0.03	0.61 ± 0.1

*Fourth- and fifth-order mesenteric arterioles characteristics are shown. Values are shown as mean ± SEM. Statistics were analyzed with two-way analysis of variance (*P* ≤ 0.05)*.

*n, number of vessels*.

*P ≤ 0.05 vs. sham control.

### Endothelium-Dependent Reactivity

**Figure 1:**
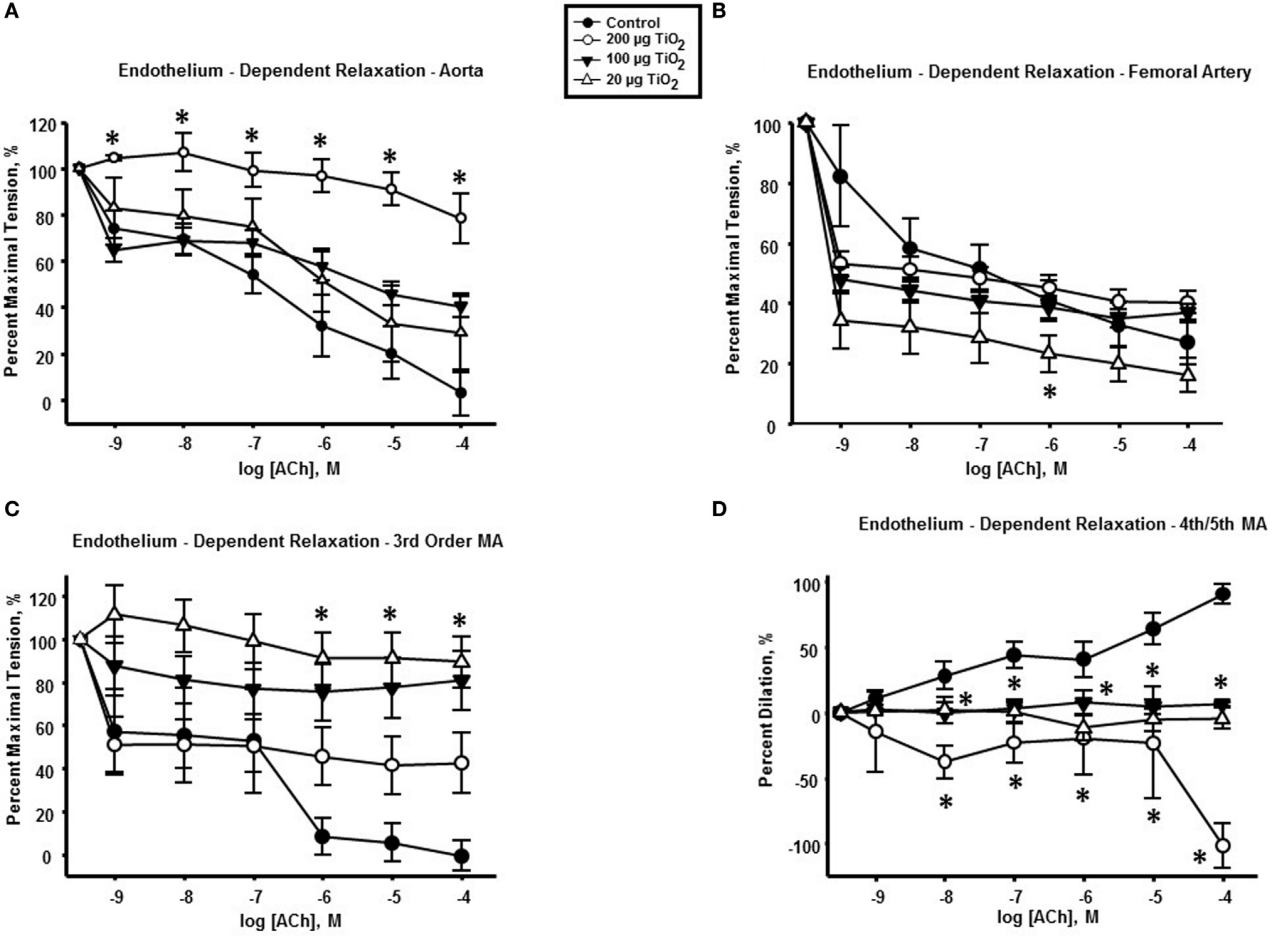
**Endothelium-dependent dilation is impaired by intratracheal instillation of nano-TiO_2_**. acetylcholine (ACh)-induced vascular reactivity in **(A)** aorta, **(B)** femoral artery, **(C)** third-order mesenteric arterioles, and **(D)** fourth- and fifth-order mesenteric arterioles (*n* = 10–11). Statistics were analyzed with two-way analysis of variance (ANOVA) (*P* ≤ 0.05). *Sham control group vs. nano-TiO_2_-exposed groups.

Two hundred micrograms of nano-TiO_2_ significantly impaired endothelium-dependent relaxation of thoracic aorta (Figure 1A) with a mean decrease in response to ACh of 53.96 ± 11.6%. No significant effects were seen at 100 and 20 µg of nano-TiO_2_ (Figure 1A).

Femoral arteries from exposed animals showed a reduced cholinergic response solely at the lowest exposure concentration (20 µg nano-TiO_2_/250 μL of vehicle) of 25.08 ± 6.36% (Figure 1B). Further, 100 and 20 µg nano-TiO_2_ exposure resulted in a mean reduction in relaxation of third-order mesenteric arterioles of 50.12 ± 8.7 and 68.28 ± 8.7%, respectively (Figure 1C). Finally, cholinergic reactivity of fourth- and fifth-order mesenteric arterioles was negatively affected by nano-TiO_2_ with diminished dilations of 82.86 ± 12.6% after exposure to 200 µg nano-TiO_2_, 42.6 ± 12.6% after 100 µg nano-TiO_2_, and 49.4 ± 12.6% after 20 µg nano-TiO_2_ (Figure 1D).

**Figure 2:**
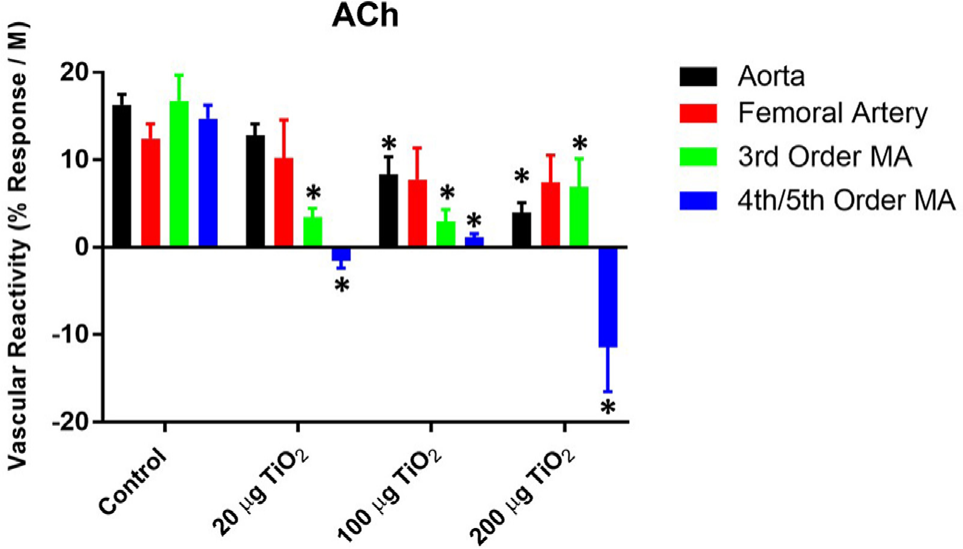
**Acetylcholine (ACh) sensitivity and vascular reactivity are decreased by nano-TiO_2_ exposure**. Bar graphs showing the individual slope values for sham control and exposed vessels (*n* = 9–11). Statistics were analyzed with two-way analysis of variance (*P* ≤ 0.05). *Sham control group vs. nano-TiO_2_-exposed groups.

To consider the fullest scope of the biological response across the discrete entities of the vasculature, the slope of the individual dose–response curves was utilized as a means of numerically quantifying vascular reactivity (Figure 2) as previously shown (7, 31). Exposure to 200 µg of nano-TiO_2_ decreased sensitivity to ACh from 16.29 ± 1.2 to 3.99 ± 1.1 M^−1^. Similarly, vascular reactivity of femoral arteries was diminished from the control value of 12.43 ± 1.7 to 7.77 ± 3.6 M^−1^ and 7.44 ± 3.1 M^−1^ with 200 and 100 µg nano-TiO_2_, respectively. Third-order mesenteric arterioles showed a dose-dependent reduction in ACh sensitivity from control levels (16.76 ± 2.9 M^−1^) with 100 µg nano-TiO_2_ having the greatest deleterious effect (2.98 ± 1.3 M^−1^), while 200 and 20 µg nano-TiO_2_ reduced responsiveness to 6.93 ± 3.2 and 3.46 ± 0.99 M^−1^, respectively. Fourth- and fifth-order mesenteric arterioles showed the greatest decrease in vascular reactivity from controls (14.73 ± 1.5 M^−1^) particularly with 200 µg nano-TiO_2_ (−11.46 ± 5.1 M^−1^) and 20 µg nano-TiO_2_ (−1.55 ± 0.8 M^−1^), while vessels exposed to 100 µg nano-TiO_2_ showed slight ACh sensitivity (1.15 ± 0.4 M^−1^). These results provide additional evidence of an endothelium-dependent effect associated with nanomaterial exposure. The heterogeneity in impairment across the vasculature indicates the microcirculation as the prime site where these effects are most markedly manifest.

### α-Adrenergic Sensitivity

**Figure 3:**
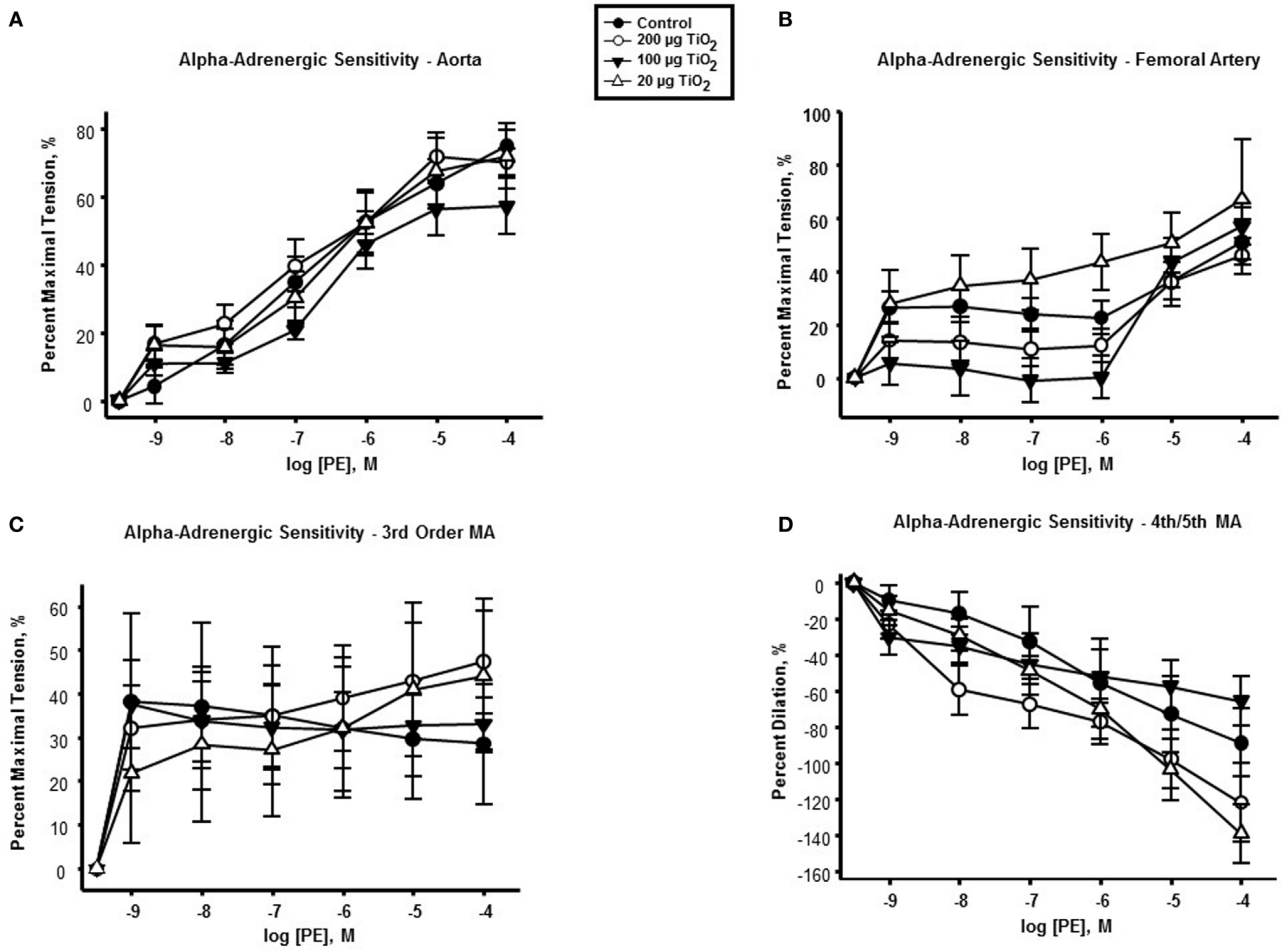
**α-adrenergic response to phenylephrine (PE) is not affected by nano-TiO_2_**. PE response in **(A)** aorta, **(B)** femoral artery, **(C)** third order mesenteric arterioles, and **(D)** fourth and fifth mesenteric arterioles (*n* = 10–11). Statistics were analyzed with two-way analysis of variance (ANOVA) (*P* ≤ 0.05).

**Figure 4:**
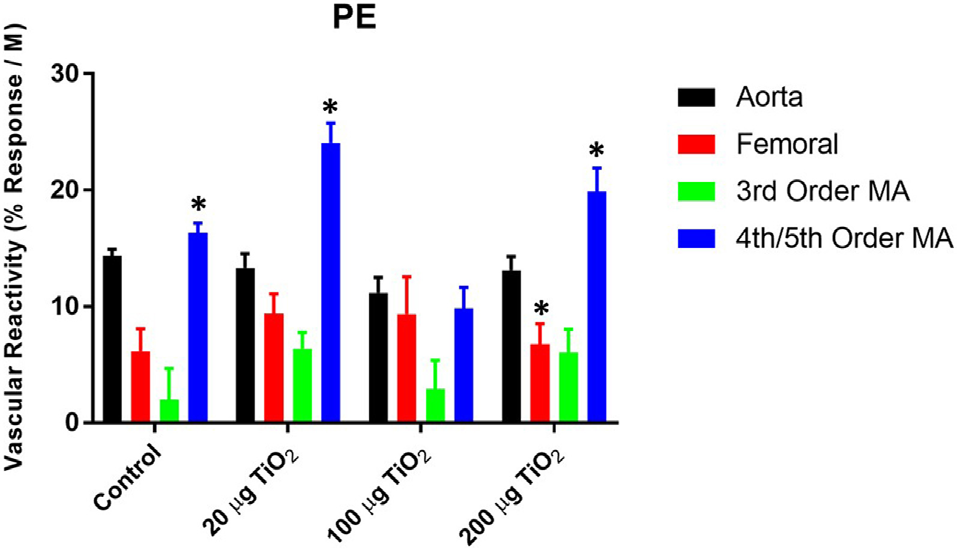
**Phenylephrine (PE) sensitivity are augmented by nano-TiO_2_ exposure**. Bar graphs showing the individual slope values for sham control and exposed vessels (*n* = 10–11). Statistics were analyzed with two-way analysis of variance (ANOVA) (*P* ≤ 0.05). *Sham control group vs. nano-TiO_2_-exposed groups.

In contrast to endothelium-dependent relaxation, no point-to-point differences were seen in α-adrenergic responses across all levels of the vasculature between nano-TiO_2_-exposed and control groups (Figures 3A–D). However, slope analysis, wherein the entire continuum of the vascular PE responses is assessed, revealed significant differences in vascular sensitivity induced by nano-TiO_2_ (Figure 4). Aortic PE sensitivity was increased by exposure to 100 µg nano-TiO_2_ (11.2 ± 1.3 M^−1^) with respect to control values (14.35 ± 0.56 M^−1^). Significantly augmented adrenergic responses were seen in the femoral artery with 100 (9.35 ± 3.2 M^−1^) and 20 µg nano-TiO_2_ (9.39 ± 1.7 M^−1^). Also, 200 µg nano-TiO_2_ (6.03 ± 2 M^−1^) and 20 µg nano-TiO_2_ (6.36 ± 1.4 M^−1^) caused increased vascular reactivity of third order mesenteric arterioles relative to controls (1.95 ± 2.7 M^−1^). Lastly, fourth and fifth mesenteric arterioles from exposed animals showed a clear proconstrictive effect particularly at 200 µg nano-TiO_2_ (19.9 ± 2 M^−1^) and 20 µg (24.07 ± 1.7 M^−1^), with 100 µg (19.9 ± 2 M^−1^) leading to responses similar to controls (16.33 ± 0.84 M^−1^) (Figure 4). This effect is consistent with previous work (26, 32) and confirms a potential effect on sympathetic tone, associated with ENM exposure.

### Endothelium-Independent Reactivity

**Figure 5:**
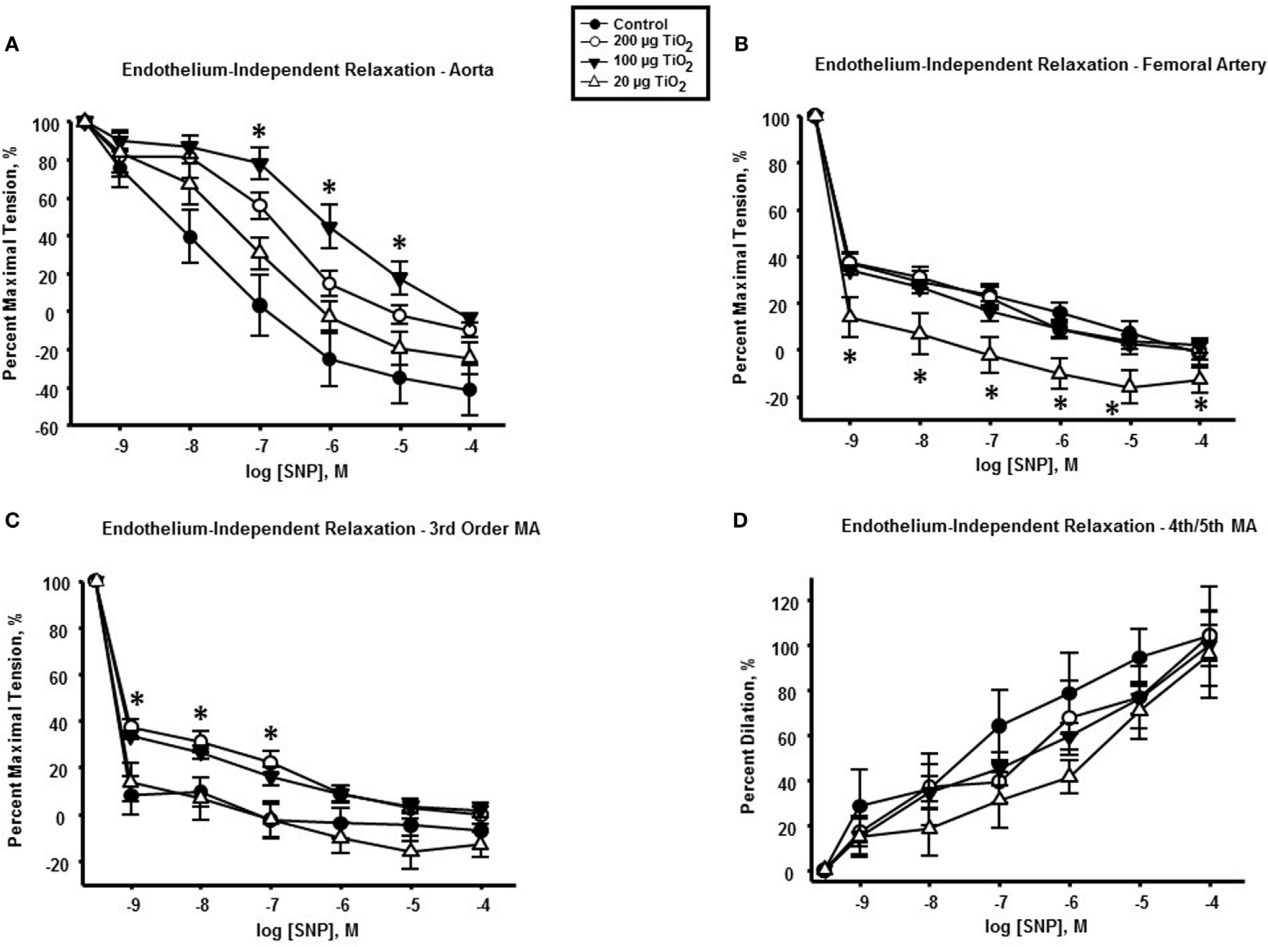
**Endothelium-independent dilation and vascular smooth muscle function is not impaired by nano-TiO_2_ exposure**. Sodium nitroprusside (SNP) response in **(A)** aorta, **(B)** femoral artery, **(C)** third-order mesenteric arterioles, and **(D)** fourth- and fifth-order mesenteric arterioles (*n* = 10–11). Statistics were analyzed with two-way analysis of variance (ANOVA) (*P* ≤ 0.05). * Sham control group vs. nano-TiO_2_-exposed groups.

**Figure 6:**
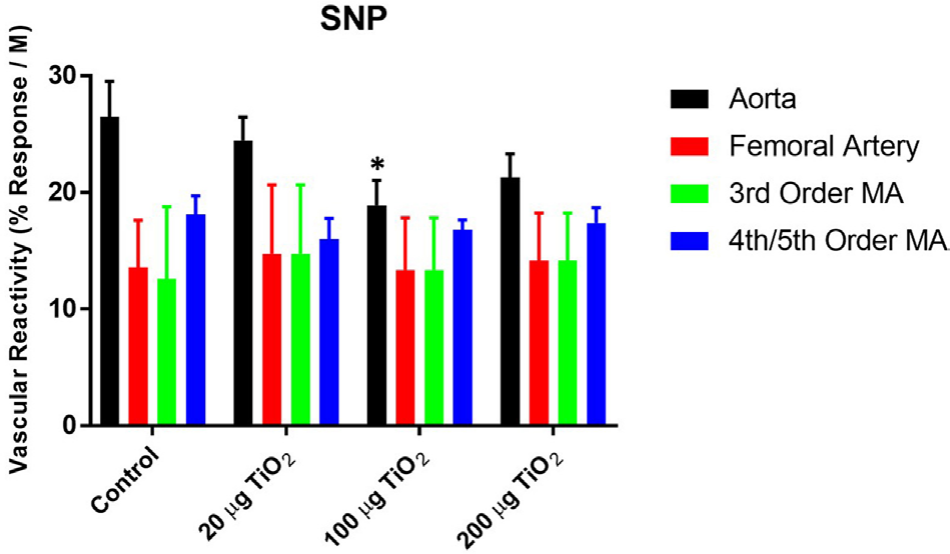
**Sodium nitroprusside (SNP)-induced vascular reactivity is unaffected by nano-TiO_2_ exposure**. Bar graphs showing the individual slope values for sham control and exposed vessels (*n* = 10–11). Statistics were analyzed with two-way analysis of variance (ANOVA) (*P* ≤ 0.05). *Sham control group vs. nano-TiO_2_-exposed groups.

Endothelium-independent relaxation assessed *via* the spontaneous NO donor SNP was impaired in the thoracic aorta with a mean decrease in response of 34.05 ± 25% induced by exposure to 200 µg nano-TiO_2_ and a reduction in response of 49.31 ± 25% caused by 100 µg nano-TiO_2_ (Figure 5A). Similarly, the response of the femoral artery to SNP was reduced by 18 ± 5% (Figure 5B). Third-order mesenteric arterioles were negatively affected by 20 µg nano-TiO_2_ with a mean decrease in response of 38.37 ± 10% (Figure 5C). It is interesting to note that slope analysis demonstrated a decrease in SNP sensitivity only in thoracic aorta at 200 µg (21.28 ± 2.03 M^−1^) and 100 µg (18.91 ± 2.1 M^−1^) nano-TiO_2_ compared to control (26.46 ± 3.08 M^−1^) (Figure 6). Similarly, fourth- and fifth-order mesenteric arteriolar sensitivity to SNP was significantly reduced from (18.13 ± 1.6 M^−1^) to (15.99 ± 1.79 M^−1^) by exposure to 20 µg nano-TiO_2_. This is consistent with previous findings where impairments in endothelium-independent dilation in the microcirculation are not noted (33–35).

## Discussion

This is the first study to directly compare the differential effect of ENM exposure on discrete anatomical segments of the vascular tree. Pulmonary ENM exposure produced macrovascular and microvascular dysfunction resulting in impaired responses to endothelium-dependent, endothelium-independent, and adrenergic agonists. The major finding of this article is that acute nano-TiO_2_ pulmonary exposure results in a more robust dysfunction at the microvascular level, and this is not simply because arterioles possess greater reactivity than arteries.

The microcirculation is critical in the regulation of the vascular internal environment for the controlled exchange of nutrients and metabolic waste with the adjacent tissues. Specifically, arterioles are major hemodynamic regulators and key players in the maintenance of blood pressure and flow distribution (13, 14). Disruptions in microvascular function are commonly associated with health consequences such as hypertension and diabetes, and this is largely due to endothelial dysfunction (36).

The pronounced effect of ENM exposure on the microcirculation may be due to the propensity of nanoparticles to accumulate preferentially in this vascular compartment. Considerable evidence exists suggesting that after the initial exposure, nanomaterials tend to translocate and accumulate systemically (37, 38). Due to the existing hydrodynamic influences present within the microcirculation, it has been hypothesized that ENM deposition would likely be highest in the arterioles (39). Within arterioles, ENM may directly impair vascular function *via* generation of free radicals, decreasing NO bioavailability, NO synthase uncoupling, or by altering sympathetic tone.

Cholinergic activity was assessed using ACh. In normal conditions, ACh activates endothelial NO synthase (eNOS) by stimulating the release of intracellular calcium. l-Arginine is converted to l-citrulline by eNOS with the production of NO. eNOS requires a number of cofactors, including NADPH, FAD, calcium, calmodulin, and BH_4_. NO subsequently diffuses into vascular smooth muscle cells and stimulates guanylate cyclase, ultimately resulting in cGMP production and vasodilation. In this study, reduced reactivity was seen in the thoracic aorta, femoral artery, third-order mesenteric arterioles and fourth-/fifth-order mesenteric arterioles. These effects are consistent with previous results obtained by our group and others (32, 40, 41). It is interesting to note, however, that exposure to 200 µg nano-TiO_2_ resulted in a complete reversal of the vasodilatory response by fourth-/fifth-order mesenteric arterioles, potentially indicating severe irreparable damage to the endothelial layer.

The endothelium is a major regulatory component common to all levels of the vasculature and plays a critical role in the control of vascular tone, the inflammatory response, maintenance and regulation of blood fluidity, permeability, and angiogenesis (42). Due to the significant role played in vascular homeostasis, it is becoming increasingly recognized that the endothelium is involved in most disease conditions, either as a primary determinant of pathophysiology or as a result of collateral damage (43).

No point-to-point impairments were seen in the response to the α-adrenergic agonist PE. However, when considering the whole continuum of the vascular responses to this agonist, a prevalent increase in sensitivity, particularly at the microvascular level, was observed (Figure 4), consistent with previous work (26, 32).

Finally, endothelium-independent dysfunction was observed primarily in the macrocirculation, including the thoracic aorta, femoral artery, and third-order mesenteric arterioles (Figures 5 and 6). This differential effect on vascular smooth muscle response in the macrocirculation may be caused by the turbulent flow patterns characteristic of this segment, which contribute to an increased likelihood of deposition and impaction of ENM agglomerates particularly at large artery bifurcations (39).

Dysfunction in vascular smooth muscle following particulate matter exposure was first reported in pulmonary arteries by Courtois et al. (44). Previous work attributed this effect to a pro-inflammatory response triggered by particulate matter exposure, while other studies linked vascular dysfunction to an increase in oxidative stress (45).

Historically, the adverse systemic effects associated with pulmonary ENM exposure have been ascribed to a host inflammatory response, direct particle–tissue interactions, and/or autonomic dysregulation (3). The observed macrovascular and microvascular dysfunction could be associated with impaired NO production, a decrease in NO bioavailability caused by the generation of reactive oxygen species within the vascular wall, a shift in arachidonic acid metabolites, or vascular smooth muscle impairment or a combination of these mechanisms. It is outside the scope of this study to determine the mechanisms underlying the vascular endpoints herein reported, and future experiments are necessary to elucidate these mechanisms.

While the results of this study provide further indications of the microvascular effects associated with ENM, it is admittedly not without its limitations. Wire myography assesses the tension generated by vascular rings under isometric tension and therefore caution should be taken when interpreting the results obtained. This is particularly so for conduit arteries, in which changes in wall tension do not explicitly translate into vasomotion. Further, the use of *in vitro* techniques to evaluate vascular reactivity eliminates neurogenic and hormonal influences, which also play an important role in the mechanism of ENM cardiovascular toxicity. Therefore, future experiments utilizing *ex vivo* preparations (such as intravital microscopy) may be more appropriate to obtain a more thorough assessment.

## Ethics Statement

All procedures were approved by the Institutional Animal Care and Use Committee of West Virginia University.

## Author Contributions

AA designed and performed the experiments, analyzed data, and wrote the paper; CM and JY provided technical support and edited the manuscript; TN and PS designed the experiments, supervised the study, and revised the manuscript.

## Conflict of Interest Statement

The authors declare that the research was conducted in the absence of any commercial or financial relationships that could be construed as a potential conflict of interest.
